# Surprising Variability in Tryptamine Profiles of *Psilocybe cubensis* Fruiting Bodies: Inter- and Intra-Strain Differences Across 14 Strains Cultivated Under Controlled Conditions

**DOI:** 10.3390/jof12070486

**Published:** 2026-07-02

**Authors:** Amiel Sharchaton, Shilat Parsha, Sara P. Azerrad, Yaron Dekel, Nisreen Rabah, Tomáš Páleníček, Eyal Kurzbaum

**Affiliations:** 1 Water Science Department, Tel-Hai Academic College, Upper Galilee 1220800, Israel; 2 Shamir Research Institute, University of Haifa, P.O.B. 97, Katzrin 1290000, Israelsaraa@gri.org.il (S.P.A.); yarond@gri.org.il (Y.D.); nis.r1993@gmail.com (N.R.); 3 The Cheryl Spencer Institute of Nursing Research, University of Haifa, Haifa 3498838, Israel; 4 Department of Evolutionary and Environmental Sciences, School of Environmental Sciences, University of Haifa, Haifa 3498838, Israel; 5 National Institute of Mental Health, 250 67 Klecany, Czech Republic; tomas.palenicek@nudz.cz; 6 3rd Faculty of Medicine, Charles University, 100 00 Prague, Czech Republic; 7 School of Environmental Sciences, University of Haifa, Haifa 3498838, Israel

**Keywords:** fungi, psilocybin, psilocin, tryptamines, *Psilocybe*, *Psilocybe cubensis*, strain, psychedelic mushrooms

## Abstract

Psilocybin-producing mushrooms exhibit considerable biochemical diversity, yet the extent of variability among strains within a single species under standardized conditions remains insufficiently characterized. In this study, we quantified psilocybin, psilocin, baeocystin, norbaeocystin, aeruginascin, and norpsilocin in the fruiting bodies of 14 distinct strains of *Psilocybe cubensis*. The mushroom strains were cultivated, dried, extracted, and analyzed under uniform laboratory conditions. Despite strict methodological standardization, total tryptamine concentrations varied by more than 7.8-fold among strains (from 2.62 to 20.65 mg/g), with psilocybin consistently emerging as the dominant compound. Analysis of individual fruiting bodies within the selected strains revealed substantial intra-strain variability, with coefficients of variation for psilocybin ranging from 12.81% to 23.39% between individual fruiting bodies. These findings demonstrate that both inter- and intra-strain biochemical heterogeneity persist even under controlled conditions, underscoring the challenges of standardizing whole mushroom preparations for research or therapeutic use. Our results highlight the importance of strain selection, rigorous chemical profiling, and dosing precision in future pharmacological and clinical applications of *P. cubensis*.

## 1. Introduction

Psychedelic mushrooms, primarily those containing psilocybin and related tryptamines, have been utilized for millennia across diverse indigenous cultures for their profound spiritual and healing properties, notably in sacred rituals and healing ceremonies. The psychoactive effects of psilocybin mushrooms are attributed mainly to tryptamines such as psilocybin and psilocin [1,2,3]. Among the various psilocybin mushroom species, *Psilocybe cubensis* stands out as one of the most widely recognized and cultivated due to its global distribution, relative ease of cultivation, and distinct morphological features [3,4]. This species, first documented in Cuba in 1906, features a cap evolving from conical–campanulate to convex and flat, pale gray gills that deepen to sepia, and a hollow, yellowish stem, facilitating its identification [5].

During the 1950s, psilocybin mushrooms attracted increasing attention for their hallucinogenic and psychotropic effects. The discovery that these fungi contain compounds structurally similar to serotonin, a neurotransmitter essential to mood regulation, prompted interest in their potential therapeutic applications, particularly for psychiatric conditions such as depression. It was also during this era that Hofmann and collaborators successfully isolated, identified, and chemically synthesized the active substances responsible for the mushrooms’ psychedelic actions: psilocybin and psilocin [6].

In recent years, with renewed interest, the frontier of mycological, biochemical, neuroscience, and pharmacological research has increasingly focused on the potential therapeutic value of psilocybin-containing mushrooms. Modern scientific investigations have convincingly demonstrated psilocybin’s efficacy in treating a range of neurological, psychiatric, and related disorders. Notably, it has shown promise for conditions such as therapy-refractory depression, major depressive disorder (MDD), and alcohol use disorder and as adjunctive therapy for tobacco dependence, anxiety, post-traumatic stress disorder (PTSD), obsessive–compulsive disorder (OCD), existential distress, anxiety, depression associated in paliative carfe, and anorexia nervosa [3,4,7,8,9,10]. In recognition of this therapeutic potential, the US Food and Drug Administration (FDA) has granted psilocybin a “Breakthrough Therapy Designation” for treatment-resistant depression and MDD.

Despite growing recognition and research interest in psilocybin-producing fungi, several knowledge gaps and challenges persist, particularly regarding variability in the concentration of psychoactive compounds such as psilocybin, psilocin, baeocystin, norbaeocystin, norpsilocin, and aeruginascin. Variation in alkaloid production is documented not only across distinct *Psilocybe* species collected from natural environments but also among strains of the same species maintained under laboratory conditions, particularly in *P. cubensis*, a species widely cultivated in both domestic and research contexts [4]. Although investigations remain limited due to legal restrictions on the possession of these fungi and their psychoactive constituents, several available reports describe marked differences (more than an orders of magnitude) in psilocybin content among strains, with measured concentrations spanning from 11.8 to 13 mg/g in the *P. cubensis* strains ‘Creeper’ and ‘Blue Meanie’ (lab grown) [11] to 1 mg/g (wild-collected) [12].

Importantly, a critical review of the literature indicates that these reported discrepancies are not attributable to genetic background alone. According to data published in the literature, even within a single strain, tryptamine levels are substantially influenced by cultivation parameters; the developmental stage at harvest; post-harvest handling, including drying and extraction procedures; and the analytical methodologies used for quantification [13,14]. Together, these findings emphasize the need for standardized experimental designs to disentangle genetic effects from environmental and methodological influences, thereby enabling a more accurate assessment of variations in the biosynthesis and accumulation of active compounds among different strains within the same species.

In this study, we quantified active tryptamine concentrations in 14 strains of *P. cubensis* under fully standardized cultivation, drying, extraction, and analytical conditions to ensure robust inter-strain comparisons. By minimizing methodological variability, we enabled a reliable assessment of strain-dependent differences in tryptamine profiles. Additionally, individual fruiting bodies were analyzed to characterize intra-strain variation, facilitating discrimination between strain-level differences and biological variability among sporocarps.

## 2. Materials and Methods

### 2.1. Mushroom Cultivation

The fungal material used in this study consisted of cultivated *P. cubensis* strains obtained from Psilution Ltd. (Katzrin, Israel) and Wombat Labs (Dallas, TX, USA). Species identity was subsequently confirmed by ITS sequencing, as described below. The strains were originally grown from spore prints or mycelial cultures and routinely maintained on potato dextrose agar (PDA) at 25 °C in the dark in our laboratory. Fruiting bodies were produced from these cultures via the following procedure. For seed spawn preparation, a mixture of wheat and millet grains (1:1) was combined with water and sterilized in autoclave bags at 121 °C for 90 min. After sterilization, the seeds were aseptically inoculated with mycelium from the PDA plates and incubated in the dark at 25 °C until complete mycelial colonization was observed (~20 days).

Fruiting bodies were produced by mixing 650 g of compressed coconut coir with 2 L of vermiculite and 4 L of boiling water in a sterilized bucket. The bucket was sealed, and the substrate was allowed to cool to room temperature before use. This procedure served as a heat pasteurization and hydration step rather than full sterilization by autoclaving. The cooled substrate was then mixed with the colonized seed spawn at a 1:2 ratio and transferred to fruiting chambers at a depth of 8 cm. The fruiting chambers, referred to as monotubs, consisted of 19 L plastic boxes measuring (20 × 35 × 27) cm^3^. Before use, the monotubs were cleaned and surface disinfected with 70% ethanol. The monotubes were incubated under a 12 h light/dark cycle at a relative humidity above 80% to induce primordia formation and subsequent fruiting body development.

**Figure 1:**
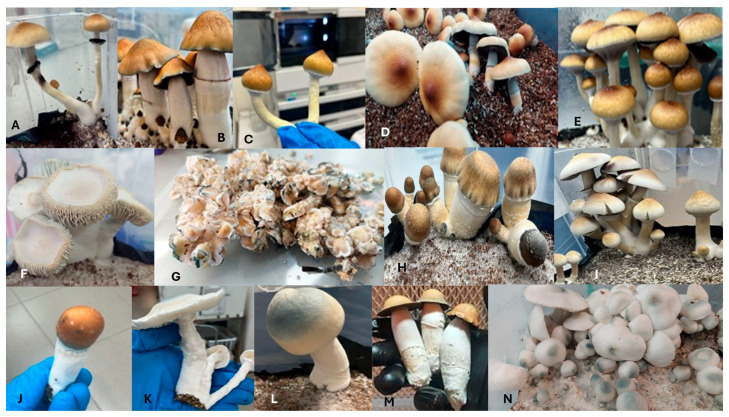
Fruiting bodies of different *P. cubensis* strains cultivated in this study. Each strain was grown separately in an individual growth chamber on a coco vermiculite substrate. All samples were cultivated, dried, and extracted under identical conditions. (**A**) “Xico”, (**B**) “Jedi Mind Fck”, (**C**) “Sat”, (**D**) “Z+”, (**E**) “Mal”, (**F**) “SE Houston”, (**G**) “Enigma”, (**H**) “Jack Frost × Golden Teacher”, (**I**) “Oak Ridge light”, (**J**) “Mak IND”, (**K**) “Jack Frost × Shakti”, (**L**) “Albino Penis Envy” (**M**) “Penis Envy”, and (**N**) “Jack Frost” (© photos by the authors).

Mature fruiting bodies (an opened veil) were morphologically and genetically examined and classified as *P. cubensis* strains according to established macromorphological criteria, including pileus shape, size, coloration, and stipe characteristics (Figure 1). Observed phenotypes were then evaluated against the declared culture designation, our prior cultivation experience, and documented strain descriptions available from public sources to verify strain identity and confirm consistency between the assigned strain name and the resulting fruiting body morphology. In the absence of a comprehensive genetic reference database for these strains, particular care was taken in the phenotypic verification process. Harvested fruiting bodies were cleaned, lyophilized (Alpha 1–2 LSCbasic, Martin Christ, Osterode am Harz, Germany), and ground into a fine powder using a mortar and pestle (Local Supplier, Israel).

### 2.2. Extraction of Tryptamines from Mushroom Material

Tryptamines were extracted following the method of Gotvaldová et al. (2021) [14]. Briefly, 100 mg (±5 mg) of the obtained dry mushroom powder was placed in a 2 mL centrifuge tube, and 1.5 mL of methanol and acetic acid (99.5:0.5, *v*/*v*) was added as an extraction solvent. The suspension was then vortexed for 10 s and incubated at 50 °C for 1 h under agitation (1800 RPM; Thermo shaker TS100, Hangzhou Ruicheng Instrument Co., Ltd., Hangzhou, China). The samples were centrifuged at 1480× *g* for 10 min, the supernatant was collected, and the pellet was subjected to a second extraction using an additional 1.5 mL of solvent as described above. The two supernatants were combined and filtered through a 0.22 μm membrane for further analysis.

### 2.3. Analytical Techniques

Psilocybin, psilocin, norbaeocystin, aeruginascin, baeocystin, and norpsilocin were quantified via high-performance liquid chromatography with photodiode array detection (HPLC–PDA) using an Agilent 1260 system (Agilent Technologies, Inc., Santa Clara, CA, USA). Chromatographic separation was performed on a reversed-phase Force Biphenyl column (Restek Restek Corporation, Bellefonte, PA, USA; 4.6 mm × 150 mm, 3 µm) maintained at 50 °C. The mobile phases were (A) water containing 0.1% (*v*/*v*) formic acid and (B) methanol containing 0.1% (*v*/*v*) formic acid. The gradient program was as follows: 5% B held for 0.6 min, increased linearly to 35% B over 7.0 min, returned to 5% B, and held for 5.0 min (total run time: 17.5 min). The flow rate was 1.5 mL/min, the injection volume was 4 µL, and PDA detection was performed at 265 nm. The limit of quantitation (LOQ) was 3 mg/L. Analytical standards were obtained from Cayman Chemical (Ann Arbor, MI, USA).

### 2.4. Experimental Setup

#### Variability in Tryptamine Profiles of *P. cubensis*: Inter- and Intra-Strain Differences

To assess inter-strain differences, defined here as variability in tryptamine content across different *P. cubensis* strains, 14 distinct *P. cubensis* strains were cultivated under identical controlled conditions. Following harvesting and drying of the fruiting bodies, as described in Section 2.1, the dried material from each strain was ground into powder. For each strain, at least three samples were extracted and analyzed from the obtained powder to generate a representative average of the tryptamine concentration (technical replicates). To assess intra-strain variations in tryptamine content among *P. cubensis* fruiting bodies of the same strain grown in the same growth chamber, the same procedure was followed for six randomly selected strains. For each strain, five randomly selected fruiting bodies (biological replicates) were harvested simultaneously from the same growth chamber at the same developmental stage to ensure comparability among samples. The fruiting bodies were then dried and extracted in triplicate, as previously described (three technical replicates per fruiting body). The coefficient of variation (CV) for the psilocybin content was determined for each strain, representing the percentage of the standard deviation (SD) relative to the mean, using the formula: CV (%) = (SD/Mean) × 100.

### 2.5. Molecular Confirmation of P. cubensis Strains by ITS Sequencing

#### 2.5.1. Fungal Material

The fourteen fungal cultures were analyzed by ITS Sequencing to confirm that all investigated strains belong to a single species, *P. cubensis.* One additional strain belonging to a different species (*Panaeolus cyanescens*) was included as a negative control. All strains were maintained as pure cultures on PDA plates, and genomic DNA was extracted from actively growing mycelium. Detailed information regarding all analyzed strains, sequence identifiers, and GenBank accession numbers is provided in Supplementary Table S1.

#### 2.5.2. DNA Extraction

Genomic DNA was extracted from actively growing mycelium collected from agar culture plates using the DNeasy Plant Mini Kit (QIAGEN, Hilden, Germany; Cat. No. 69104), according to the manufacturer’s instructions.

#### 2.5.3. PCR Amplification of ITS Regions

The internal transcribed spacer (ITS) regions were amplified using universal fungal primers ITS1_F, ITS2_R, ITS3_F, and ITS4_R, originally described by [15]. Primer sequences were as follows: ITS1_F (5′-TCCGTAGGTGAACCTGCGG-3′), ITS2_R (5′-GCTGCGTTCTTCATCGATGC-3′), ITS3_F (5′-GCATCGATGAAGAACGCAGC-3’), and ITS4_R (5′-TCCTCCGCTTATTGATATGC-3′).

PCR amplification was performed using HY-TIGER 2× PCR Master Mix (EZ-2031HIGH, HyLabs Ltd., Israel) according to the manufacturer’s instructions. Reactions were carried out in a total volume of 50 µL.

Amplification of the ITS1 region was performed using primers ITS1_F/ITS2_R, while the ITS2 region was amplified using ITS3_F/ITS4_R.

Thermal cycling conditions consisted of an initial denaturation at 95 °C, followed by 30–35 cycles of denaturation at 95 °C for 30 s, annealing at 59 °C for ITS1 and 55 °C for ITS2 for 30 s, and extension at 72 °C for 1 min, with a final extension at 72 °C for 5–10 min.

#### 2.5.4. Sequencing and Data Analysis

PCR products were analyzed using the QIAxcel Advanced system (QIAGEN), allowing high-resolution fragment separation and verification of amplicon size and specificity. PCR products were subsequently submitted for purification and Sanger sequencing.

The obtained sequences were analyzed using BLAST(^®^) (blastn suite, (NCBI)) for species identification, and multiple sequence alignments were performed using T-COFFEE to assess sequence similarity and variation among strains. To ensure accurate delimitation of ITS subregions, sequence boundaries were defined according to the annotated reference sequence of *P. cubensis* (GenBank accession AY281021.1).

## 3. Results

### 3.1. Tryptamine Content Variability Across 14 P. cubensis Strains Cultivated Under Identical Controlled Conditions

Table 1 presents the concentrations of tryptamine compounds (psilocybin, psilocin, norpsilocin, baeocystin, norbaeocystin, and aeruginascin) measured in various *P. cubensis* strains, which were grown, dried, extracted, and analyzed under identical and standardized laboratory conditions. Despite this strict standardization, we observed pronounced inter-strain differences in the tryptamine content. The total indole alkaloid concentrations varied by more than sevenfold, ranging from 20.65 mg/g in the “Mak IND” strain to 2.62 mg/g in “Oak Ridge light”. Psilocybin was the dominant compound in all strains and showed a wide range, varying from 19.60 ± 2.93 mg/g in “Mak IND” to 2.03 ± 0.56 mg/g in “Oak Ridge light.” Other tryptamines also displayed clear strain-specific patterns. Psilocin ranged from 1.77 ± 0.14 mg/g in “Penis Envy” to 0.11 ± 0.03 mg/g in “Enigma”, while baeocystin ranged from 0.43 ± 0.04 mg/g in “Sat” to below the limit of detection (<LOD) in others. Other tryptamines, such as norbaeocystin and aeruginascin, were detected only at low concentrations and frequently below the LOD.

Together, these results reveal marked strain-level variability in both the total amounts and relative proportions of psychoactive tryptamines. No consistent relationship was detected among the different compounds, as high levels of one did not predict higher or lower levels of the others, showing that the biosynthesis of individual tryptamines is not tightly coordinated across strains. Consequently, species-level identification alone is insufficient to predict psychoactive profiles, and strain-specific factors appear to play a dominant role in determining both compound concentration and composition.

### 3.2. Intra-Strain Variability in Tryptamine Content Among Individual Fruiting Bodies Across Selected P. cubensis Strains

To quantify the intraspecific variation observed among five individual fruiting bodies per strain of *P. cubensis*, coefficients of variation (CVs) were calculated for the psilocybin concentration within each of the six examined strains. Table 2 shows that the CV values revealed pronounced heterogeneity among individual fruiting bodies, even though all samples originated from the same genetic organism and were cultivated within the same growth chamber under identical cultivation and processing conditions. Notably, the strain Jack Frost × Shakti exhibited the highest CV of approximately 23.39%. This example illustrates that intra-strain biochemical variability can be considerable and, in some cases, dominate over experimental or analytical sources of variation, highlighting the limitations of single-specimen-based assessments, such as sampling a single carpophore, when characterizing strain-level psychoactive profiles.

### 3.3. Species Confirmation of P. cubensis Strains by ITS Sequencing

Molecular identification of all studied strains was performed using ITS1 and ITS2 sequence analyses, which are widely accepted as standard DNA barcoding markers for fungal taxonomy.

A total of fifteen fungal cultures were analyzed, including fourteen strains presumed to belong to *P. cubensis* and one strain (*Panaeolus cyanescens*) used as a negative control. Sequence similarity analyses were conducted for both ITS1 and ITS2 regions.

All sequences generated in this study were deposited in GenBank under accession numbers PZ374629-PZ374643, PZ374693-PZ374707, PZ374729, and PZ374730. Detailed strain information, sequence identifiers, and corresponding GenBank accession numbers are provided in Supplementary Table S1. Sequence similarity percentages and query coverage values obtained from BLAST analyses for both ITS1 and ITS2 regions are summarized in Supplementary Table S2.

Analysis of the ITS1 region revealed a high degree of sequence conservation among all fourteen *P. cubensis* strains. Pairwise sequence identity values ranged from 97.78% to 99.10%, with query coverage values between 97% and 100%. In contrast, the negative control exhibited substantially lower similarity, with 75.81% identity and 25% query coverage, indicating clear divergence from the *P. cubensis* group.

Analysis of the ITS2 region further supported these findings. Thirteen of the fourteen strains showed complete sequence identity (100%), while one strain (Jack Frost) displayed minor variation (99.42%), consistent with intraspecific variability. All strains exhibited 100% query coverage. In contrast, the negative control showed significantly lower similarity (73.72% identity and 90% query coverage), confirming its distinct taxonomic position. BLASTn analyses of both ITS1 and ITS2 sequences obtained from the negative control further confirmed its taxonomic identity as *Panaeolus cyanescens*. The ITS1 sequence showed 100% sequence identity with 98% query coverage to multiple *Panaeolus cyanescens* ITS reference sequences available in GenBank, including OK643762.1 and OP862802.1, while the deposited sequence generated in this study received the GenBank accession number PZ374729. The ITS2 sequence showed 100% sequence identity and 100% query coverage to multiple *Panaeolus cyanescens* reference sequences, including HM035085.1 and AB158633.1, and the deposited ITS2 sequence generated in this study received the GenBank accession number PZ374730.

These results demonstrate a clear molecular separation between the studied strains and the negative control across both ITS regions. The high sequence similarity observed among the fourteen *P. cubensis* strains, together with low intraspecific variability and pronounced interspecific divergence, provides strong molecular evidence that all investigated strains belong to the same species, *P. cubensis*.

## 4. Discussion

This study demonstrates pronounced inter- and intra-strain differences in the tryptamine content among distinct *P. cubensis* strains. Despite strict standardization of cultivation, extraction, and analytical procedures, total tryptamine levels varied by up to 7.8-fold across strains. These findings emphasize that species and strain identification alone cannot predict the psychoactive content. To the best of our knowledge, this is the first study to provide a comprehensive, methodologically controlled comparison of multiple *P. cubensis* strains in an academic context.

Despite strict experimental standardization, we observed pronounced inter-strain differences in the tryptamine content. As shown in Table 1, tryptamine concentrations range from about 2.62 mg/g in “Oak Ridge light” to approximately 20.65 mg/g in the “Mak IND” strain. The tryptamine concentration in the “Mak IND” strain is higher than previously reported for other *P. cubensis* strains [4,11,12]. Psilocybin was the dominant compound in all strains but varied substantially in concentration. Other tryptamines (psilocin, baeocystin, norbaeocystin, and aeruginascin) also showed clear strain-specific patterns; for example, there was a higher psilocin content in “Penis Envy” than in “Enigma” and a higher baeocystin content in “Sat” than in “Jedi Mind Fck”. Taken together, these findings demonstrate substantial strain-level heterogeneity in both the absolute amounts and relative proportions of psychoactive tryptamines.

These results should be interpreted considering the substantial methodological heterogeneity in the existing literature on psilocybin-containing mushrooms. Previous studies are inconsistent due to methodological differences (e.g., growth conditions, drying, solvents, extraction time, temperature, analytical instruments), making comparison difficult [13,14,16,17]. Standardized analyses, as in this study, provide a baseline for quantifying strain-level biochemical heterogeneity. By applying the same standardized protocol across 14 *P. cubensis* strains and simultaneously measuring several tryptamines in each sample, this study provides a more internally consistent baseline for characterizing strain-level biochemical heterogeneity.

The differences between strains reported in Table 1 and Table 2 likely reflect heterogeneity in fruiting bodies rather than analytical inconsistency. Table 1 reports replicate measurements from a single prepared powder sample composed of several fruiting bodies per strain, whereas Table 2 presents measurements from five individual fruiting bodies per strain and therefore captures within-strain variability. Because sporocarp-to-sporocarp variation can be substantial even under controlled conditions, the estimated mean can shift depending on which fruiting bodies are sampled. Thus, Table 2 quantifies within-strain dispersion, while Table 1 provides standardized strain-level comparisons across strains.

From a pharmacological perspective, the observed intraspecies heterogeneity indicates that dosing based solely on biomass weight may result in inconsistent pharmacological exposure, particularly when whole-mushroom preparations are administered to patients in clinical settings, underscoring the need for rigorous chemical profiling in both research and therapeutic contexts. In addition, strain-specific minor tryptamines (psilocin, norpsilocin, baeocystin, norbaeocystin, and aeruginascin) are particularly relevant to ongoing discussions about a potential entourage effect. Emerging evidence suggests that natural mushroom extracts may produce stronger or more prolonged effects than equivalent doses of synthetic psilocybin, possibly because accompanying indole alkaloids and other compounds, e.g., the betacarbolines, modulate or amplify the action of the primary compound [18,19]. Our findings demonstrate that strains differ not only in the total psilocybin content but also in the relative proportions of other tryptamines supporting the plausibility of multi-compound interactions.

To date, most clinical trials investigating psilocybin have utilized pure synthetic formulations, such as those from Compass Pathways [20] or the Usona Institute [21]. Only a few have tested naturally sourced fungal extracts, e.g., developed by Filament Health [22]. Currently, there is only one published human study directly comparing these forms; it found that while synthetic psilocybin was perceived as less “natural” and slightly inferior in overall experiential quality, no major clinical differences were observed [23].

Overall, accumulating data indicate that therapeutic efficacy heavily relies on reaching a peak or “mystical experience,” pointing to the importance of the dosing to the treatment. Clinical dosing of psilocybin spans a broad spectrum: sub-perceptual microdoses (around 2.5 mg), low doses (5–15 mg), standard therapeutic doses (20–25 mg), high doses (25–30 mg), and supra-therapeutic doses (above 35 mg) used to evaluate safety thresholds [24]. For depression and anxiety, trials typically use 15 to 30 mg, with 25 mg being the most frequent choice because it reliably induces a full psychedelic state with minimal side effects [20,25]. Robust dose–response studies remain scarce, but pooled data underscore that the absolute dose level is paramount for achieving a therapeutic outcome, with durability ranging from several months to a year [25].

In contrast to controlled clinical trials, traditional or ritual mushroom use generally involves 1 to 5 g of dried material, with an average of approximately 2.5 to 3 g [24]. Although real-world potency varies among strains and cultivation methods, commonly used species such as *P. cubensis* and wild wood decomposing species, including *P. cyanescens* and *P. azurescens*, contain approximately 1% psilocybin by dried mass on average [26]. Thus, 2.5 to 3 g of dried fruiting bodies would correspond approximately to 25 to 30 mg of psilocybin, which is comparable to the 25 mg dose commonly used in clinical studies. Interestingly, the average psilocybin concentration across the 14 strains examined in the present study was approximately 1%, equivalent to about 10 mg/g in dried material.

Although all *P. cubensis* strains in this study were cultivated under a single standardized protocol at 25 °C using the same substrate formulation, these conditions may not represent optimal growth conditions for all strains. Future studies should therefore investigate strain-specific optimal cultivation parameters and evaluate how variations in substrate composition, temperature, humidity, and container volume influence the resulting profiles of psychoactive compounds.

Future studies may also study the production of psilocybin under submerged fermentation. Although it is assumed that submerged fermentation of mycelial cultures represents a promising approach to reducing the pronounced variability in the tryptamine content observed among individual fruiting bodies, both within and between strains, quantitative and comparative studies of *Psilocybe* spp. indicated that tryptamine alkaloids are concentrated predominantly in fruiting bodies rather than in mycelium. In a recent metabolomic analysis of *P. cubensis*, the mean psilocybin concentration was 9.913 mg/g (±0.389 mg/g) in fruiting bodies, compared with 0.041 mg/g (±0.014 mg/g) in mycelium and 0.047 mg/g (±0.023 mg/g) in grain mycelium, demonstrating a markedly greater accumulation of psilocybin in fruiting tissues [27]. Additionally, in psilocybe “truffles” (sclerotia) between 0.06% and 0.16% were reported [28]. In addition, the study by [27,29] showed that fruiting body material and mycelial material differ not only in absolute alkaloid concentration but also in overall metabolite composition, reinforcing the view that developmental stage is a major determinant of tryptamine abundance in *Psilocybe* spp.

However, a much earlier work by [30] showed that *P. cubensis* mycelium grown in submerged culture (in flasks) was nevertheless capable of synthesizing psilocybin, reaching a maximum concentration of 0.52% of dry weight on day 7 and up to 1.03% of dry weight under elevated glucose conditions, while psilocin was not detected and psilocybin was absent from the culture broth. Thus, interesting future research may focus on the cultivation of psilocybin fungi in controlled bioreactor systems. This may enable scalable production of more homogeneous biomass with improved reproducibility while enabling precise regulation of key environmental parameters such as pH, carbon and nitrogen availability, aeration, and mixing. Such controlled systems could facilitate consistent metabolite production and provide a more standardized platform for both research and potential pharmaceutical applications.

## 5. Conclusions

Even under identical cultivation and analytical conditions, *P. cubensis* strains exhibited pronounced variability in their tryptamine profiles, with more than sevenfold differences observed between strains and substantial heterogeneity among individual fruiting bodies of the same strain. These findings demonstrate that species identification alone cannot reliably predict the psychoactive compound content and highlight the importance of strain selection and chemical characterization. From a pharmacological perspective, this variability suggests that dosing based solely on mushroom biomass may lead to inconsistent pharmacological exposure when whole-mushroom preparations are used. Consequently, standardized chemical profiling and improved cultivation strategies will be essential for future research and potential therapeutic applications.

## Figures and Tables

**Table 1:** Concentrations of tryptamines (mg/g) in *P. cubensis* strains grown in the laboratory under identical controlled conditions.

*P. cubensis* Strain Name	Total Tryptamines mg/g	Psilocybin mg/g	Psilocin mg/g	Baeocystin mg/g	Aeruginascin mg/g	Norpsilocin mg/g	Norbaeocystin mg/g
Mak IND	20.65	19.60 ± 2.93	0.65 ± 0.38	0.40 ± 0.10	<LOD	<LOD	<LOD
Penis Envy	17.59	15.49 ± 1.08	1.77 ± 0.14	0.20 ± 0.006	<LOD	<LOD	0.12 ± 0.01
Sat	14.36	13.53 ± 2.96	0.38 ± 0.17	0.43 ± 0.04	<LOD	<LOD	<LOD
Jedi Mind Fck	12.70	11.58 ± 1.71	0.99 ± 0.20	0.11 ± 0.03	<LOD	<LOD	<LOD
Albino Penis Envy	12.33	11.38 ± 2.01	0.67 ± 0.30	0.18 ± 0.04	0.08 ± 0.04	<LOD	<LOD
Jack Frost × Golden Teacher	11.38	11.03 ± 1.52	0.15 ± 0.05	0.13 ± 0.04	0.06 ± 0.03	<LOD	<LOD
Enigma	8.11	7.91 ± 0.87	0.11 ± 0.03	<LOD	0.08 ± 0.05	<LOD	<LOD
Jack Frost × Shakti	7.29	6.75 ± 1.73	0.40 ± 0.22	0.12 ± 0.02	0.02 ± 0.04	<LOD	<LOD
Xico	7.10	6.22 ± 1.73	0.65 ± 0.36	0.22 ± 0.09	<LOD	<LOD	<LOD
Mal	6.34	5.62 ± 0.36	0.38 ± 0.25	0.32 ± 0.06	<LOD	<LOD	<LOD
Jack Frost	4.95	4.95 ± 0.05	<LOD	<LOD	<LOD	<LOD	<LOD
Z+	3.24	3.24 ± 0.18	<LOD	<LOD	<LOD	<LOD	<LOD
SE Houston	2.97	2.28 ± 0.60	0.68 ± 0.14	<LOD	<LOD	<LOD	<LOD
Oak Ridge light	2.62	2.03 ± 0.56	0.49 ± 0.23	0.09 ± 0.03	<LOD	0.01 ± 0.02	<LOD

LOD—limit of detection. × indicates breeding lines between two distinct cultivated strains.

**Table 2:** Quantitative analysis of intra-strain variation in psilocybin content across six *P. cubensis* strains cultivated and extracted under standardized laboratory conditions. For each strain, five individual fruiting bodies were dried and analyzed separately.

*P. cubensis* Strain Name	Fruiting Body #1 (mg/g)	Fruiting Body #2 (mg/g)	Fruiting Body #3 (mg/g)	Fruiting Body #4 (mg/g)	Fruiting Body #5 (mg/g)	Average of Fruiting Bodies (mg/g)	Standard Deviation of Averages	CV (%)
Oak Ridge Light	2.06	2.04	1.99	1.38	2.10	1.91	0.30	15.73
Xico	4.09	6.56	6.02	4.82	6.44	5.59	1.08	19.42
Jack Frost × Shakti	5.41	5.16	5.30	8.35	7.55	6.35	1.49	23.39
Jack Frost × Golden Teacher	10.29	12.38	8.98	12.22	6.81	10.10	2.29	22.70
Mal	3.92	3.43	4.44	4.78	3.91	4.10	0.52	12.81
Sat	6.03	4.99	8.66	5.60	6.89	6.43	1.42	22.13

× indicates breeding lines between two distinct cultivated strains.

## Data Availability

The original contributions presented in the study are included in the article; further inquiries can be directed to the corresponding author.

## Supplementary Materials

The following supporting information can be downloaded at: https://www.mdpi.com/article/10.3390/jof12070486/s1, Supplementary Table S1: Sequence metadata and GenBank accession numbers; Supplementary Table S2: ITS sequence similarity and BLAST identification statistics.
