# Supplementary material for: Surprising Variability in Tryptamine Profiles of Psilocybe cubensis Fruiting Bodies: Inter- and Intra-Strain Differences Across 14 Strains Cultivated Under Controlled Conditions
Source: J Fungi (Basel). 2026 Jul 2;12(7):486. doi: 10.3390/jof12070486 (PMC13412672; doi:10.3390/jof12070486)
Supplement: Supplementary file 1 [file jof-12-00486-s001.zip › jof-4223984-supplementary.pdf]

Supplementary material

Table S1. Sequence metadata and GenBank accession numbers

**name / strain**	**species**	**ITS 1**		**ITS 2**	
**name / strain**	**species**	**Sequence_ID**	**GenBank accession Numbers Sequence**	**Sequence_ID**	**GenBank accession Numbers Sequence**
Sat	*Psilocybe cubensis*	cubensis_Sat_ITS1	PZ374629	Sat_ITS2	PZ374693
Mal	*Psilocybe cubensis*	cubensis_Mal_ITS1	PZ374630	Mal_ITS2	PZ374694
Enigma	*Psilocybe cubensis*	cubensis_Enigma_ITS1	PZ374631	Enigma_ITS2	PZ374695
Jack Frost	*Psilocybe cubensis*	cubensis_Jack_Frost_ITS1	PZ374633	Jack_Frost_ITS2	PZ374697
JMF x Golden Teacher	*Psilocybe cubensis*	cubensis_JMF_x_Golden_Teacher_ITS1	PZ374634	JMF_x_	PZ374698
Jedi Mind Fck	*Psilocybe cubensis*	cubensis_Jedi_Mind_Fck_ITS1	PZ374635	Jedi_Mind_Fck_ITS2	PZ374699
Jack Frost x Shakti f7	*Psilocybe cubensis*	cubensis_Jack_Frost_x_Shakti_f7_ITS1	PZ374636	Jack_Frost	PZ374700
SE Houston	*Psilocybe cubensis*	cubensis_SE_Houston_ITS1	PZ374637	SE_Houston_ITS2	PZ374701
Mak IND	*Psilocybe cubensis*	cubensis_Mak_IND_ITS1	PZ374638	Mak_IND_ITS2	PZ374702
Penis envy	*Psilocybe cubensis*	cubensis_Penis_envy_ITS1	PZ374639	Penis_envy_ITS2	PZ374703
Xico	*Psilocybe cubensis*	cubensis_Xico_ITS1	PZ374640	Xico_ITS2	PZ374704
Albino Penis Envy	*Psilocybe cubensis*	cubensis_Albino_Penis_Envy_ITS1	PZ374641	Albino_Penis_Envy_ITS2	PZ374705
Oak Ridge light	*Psilocybe cubensis*	cubensis_Oak_Ridge_light_ITS1	PZ374642	Oak_Ridge_light_ITS2	PZ374706
Z+	*Psilocybe cubensis*	cubensis_Z+_ITS1	PZ374643	Z+_ITS2	PZ374707
Panaeolus strain	*Panaeolus cyanescens*	Panaeolus_Sason_ITS1	PZ374729	Panaeolus_Sason_ITS2	PZ374730

Supplementary Table S2. ITS sequence similarity and BLAST identification statistics

Strain	Species	ITS1		ITS2	
		Identity (%)	Cover (%)	Identity (%)	Cover (%)
Sat	*Psilocybe cubensis*	*97.78*	100	*100*	100
Mal	*Psilocybe cubensis*	*99.08*	97	*100*	100
Enigma	*Psilocybe cubensis*	*99.1*	98	*100*	100
Jack Frost	*Psilocybe cubensis*	*99.08*	97	*99.42*	100
JMF x Golden Teacher	*Psilocybe cubensis*	*99.08*	97	*100*	100
Jedi Mind Fck	*Psilocybe cubensis*	*99.08*	97	*100*	100
Jack Frost x Shakti f7	*Psilocybe cubensis*	*99.08*	97	*100*	100
SE Houston	*Psilocybe cubensis*	*99.08*	97	*100*	100
Mak IND	*Psilocybe cubensis*	*99.08*	97	*100*	100
Penis envy	*Psilocybe cubensis*	*99.08*	97	*100*	100
Xico	*Psilocybe cubensis*	*99.08*	97	*100*	100
Albino Penis Envy	*Psilocybe cubensis*	*99.08*	97	*100*	100
Oak Ridge light	*Psilocybe cubensis*	*99.08*	97	*100*	100
Z+	*Psilocybe cubensis*	*99.08*	97	*100*	100
Panaeolus strain *	*Panaeolus cyanescens*	*75.81*	*25*	*73.72*	*90*

* Negative control
